# Cyclosporine A alters expression of renal microRNAs: New insights into calcineurin inhibitor nephrotoxicity

**DOI:** 10.1371/journal.pone.0175242

**Published:** 2017-04-17

**Authors:** Jennifer L. Gooch, Clayton King, Cynthia E. Francis, Paul S. Garcia, Yun Bai

**Affiliations:** 1 Department of Pharmaceutical Sciences, School of Pharmacy, Philadelphia College of Osteopathic Medicine, Suwanee, Georgia, United States of America; 2 Department of Anesthesiology, School of Medicine, Emory University, Atlanta, Georgia, United States of America; University of California Los Angeles, UNITED STATES

## Abstract

Calcineurin inhibitors are powerful immunosuppressants that revolutionized organ transplantation. However, non-immune effects of the calcineurin inhibitor, such as cyclosporine A (CsA), have significantly hindered their use. Specifically, nephrotoxicity, which is associated with tubulointerstitial fibrosis, inflammation, and podocyte damage, affects up to half of all transplant patients. Calcineurin is involved in many aspects of kidney development and function; therefore, mechanisms of CsA-induced nephrotoxicity are complex and not yet fully understood. MicroRNAs are short non-coding RNAs that regulate protein-coding RNA expression through post-translational repression of target messenger RNAs. MicroRNA dysregulation is known to be involved in kidney diseases including fibrosis. In this study, we compared the renal microRNA expression profiles between mice that received CsA (20 mg/kg) or vehicle daily for six weeks. The results demonstrate that CsA induces significant changes in renal microRNA expression profile. We used combined criteria of False Discovery Rate (≤0.1), fold change (≥2) and median signal strength (≥50) and identified 76 differencially expressed microRNAs. This approach identified microRNAs previously linked to renal fibrosis that includes let-7d, miR-21, miR-29, miR-30, miR-130, miR-192, and miR-200 as well as microRNAs that have not been reported to be related to nephrotoxicity or immunosuppression. Pathway analysis of microRNA/mRNA changes highlights the Wnt, TGF-β, mTOR, and VEGF pathways. The mRNA expression profiles were compared in the same samples. The change of mRNA and microRNA profiles showed close correlations. To validate that the observed microRNA and mRNA expression level changes in mice kidney tissue were directly related to CsA treatment, the expression change induced by CsA treatment of three microRNAs (miR-21, miR-186, and miR-709) and three mRNAs (BMPR1a, SMURF1 and SMAD7) were compared in HEK293 cell line. A similar trend of expression level change was induced by CsA treatment in all selected microRNAs and mRNAs in the *in vitro* cell model. These data provide a roadmap for future work to study the role of the known and novel candidate microRNAs in the mechanism of nephrotoxicity and their further therapeutic potential.

## Introduction

Cyclosporine A (CsA) and the related compound tacrolimus (Tac) are the cornerstones of immunosuppressant regimens in organ transplantations and autoimmune diseases. Despite significant improvements in morbidity and mortality following their introduction [1, 2], transplant patients treated with CsA or Tac frequently suffer from a range of side effects including cardiovascular disease and nephrotoxicity [3]. Acute and chronic kidney dysfunction after transplantation is one of the most common and serious postoperative complications [4]. For example, the incidence rate of acute kidney dysfunction in heart transplant recipients can be as high as 70%[3]; 5–15% will eventually need renal replacement therapy [5]. The immunosuppressive effect of CsA and Tac is based on the inhibition of calcineurin, a calcium-dependent phosphatase required for the production of cytokines following T-cell receptor activation[6]. While calcineurin is widely expressed, the mechanism of CsA nephrotoxicity remains poorly understood, and there are currently no treatments to address the loss of kidney function.

MicroRNAs are small (usually 18–22 nucleotides), endogenous non-coding RNA oligos that can regulate gene expression of target mRNAs [7, 8]. MicroRNAs induce gene silencing or down-regulation by binding to their targeted mRNAs with complete or partially complementary sequences. Over 2000 microRNAs have been characterized in humans. They participate in the regulation of a wide range of biological processes including differentiation, proliferation, and apoptosis [9]. Recent studies have suggested that microRNAs may be biomarkers of acute kidney injury [10]. For example, increased expression of miR-21 is associated with kidney fibrosis [11–13]. MiR-21 also has been proven to be related to the rate of kidney function decline [14] and specificly associated with cyclosporine-mediated allograft fibrosis [15] which is a hallmark of the end-stage renal disease. Also, several microRNAs, such as miR-130, miR-192 and miR-200, have been reported to have a regulatory role in kidney diseases [16, 17]. Two microRNAs—miR-19 and miR-574 have been reported to be related to CsA immunosuppression effect. miR-19 has been reported to be downregulated by CsA *in vitro[18]* while miR-574 has been reported to be downregulated with acute rejection in human [19]. MiR-15 has been reported to be lower in urine with glomerulosclerosis. In addition, let-7d-3p [20], miR-21[21], miR-29 [22], miR-30 [23], miR-130 [16], miR-192 [24], miR-200 [25, 26] have been reported to be related to kidney fibrosis.

Despite recent advances, there is a gap in understanding of the role of microRNAs in nephrotoxicity induced by calcineurin inhibitors. This study aimed to identify the potential key microRNAs in the nephrotoxicity induced by CsA to deepen the understanding of the pathophysiology. We compared the renal microRNA and mRNA expression profiles in mice treated with CsA for six weeks with the same expression profiles in control mice. Our results demonstrate that 76 microRNAs are differentially expressed. Parallel analysis of mRNA expression profiling indicate that 268 genes are significantly regulated. The differentially expressed microRNAs that are significant are enriched in different cellular pathways which include PI3K-Akt, MAPK, TGF-β,Wnt, and VEGF pathways. The same trend of change in expression level of three selected microRNAs and three selected mRNAs are observed after CsA treatment in the HEK 293 *in vitro* cell model which confirms that the observed change of expressions of microRNA and mRNA are directly related to CsA treatment.

## Materials and methods

### Animal model

Six- to eight- week old male C57BL/6 mice were purchased from Charles River. All mice were maintained at the Atlanta VA Medical Center animal facility and all procedures were first approved by the Atlanta VAMC IACUC. Mice were socially housed in standard shoe box cages with a regular 12 hr light/dark cycle and fed standard rodent chow for two weeks before the experiment. Twelve (12) mice were randomly assigned to treatment or control groups. Mice in the treatment group (N = 6) received CsA (20 mg/kg) which was mixed with peanut oil and applied to moistened standard rodent chow daily for six weeks before sacrifice. Control mice (N = 6) received the peanut oil vehicle only. At the end of the study, mice were sacrificed and kidneys were harvested.

### Sample collection

All kidney samples were collected in an identical manner. One-half of each kidney was snap frozen in liquid nitrogen and then stored at -80°C. This method of freezing and storage is sufficient to preserve RNA for several years. The other half kidney portion was immediately immersed in formalin for histological analyses. Formalin-fixed sections were embedded in paraffin and sectioned at four(4) μm and then stained with hematoxylin, eosin and Trichome for routine histology.

### Immunohistochemistry (IHC)

Immunohistochemical study was performed on an automated immunostainer (Benchmark XT) with the XT Ultra view diaminobenzidine kit. Antigen retrieval was achieved by overnight incubation at 60°C in 0.1MTris/HCl buffer (pH9.0). Then, samples were incubated with the primary antibodies. Primary antibody binding was detected by sequential incubations with HRP-labeled appropriate secondary and tertiary antibodies (all obtained from DAKO). Peroxidase activity was visualized using 3,39-diaminobenzidine tetrahydrochloride (DAKO) as chromogen (10 min incubation). Negative controls were performed by omitting primary antibodies.

### Western blotting

Total protein was extracted with a modified Radio-Immunoprecipitation Assay (RIPA) lysis buffer. Protein concentrations were measured using the BCA protein assay kit (Pierce, Rockford, IL, USA). Proteins were separated by 10% SDS–PAGE gel and transferred to polyvinylidene difluoride membrane. Proteins were recognized by the primary antibody against collagen IV and β actin (loading control). Odyssey secondary antibodies were added according to manufacturers instructions (Goat anti-rabbit IRDye 680 and Goat anti-mouse IRDye 800). Blots were imaged using an Odyssey Infrared Imaging System (Li-COR Biosciences). A scan resolution of the instrument ranges from 21–339μm and, in this study, blots were imaged at 169μm. Quantification was performed on single channels with the analysis software provided.

### RNA isolation and microRNA array

The total RNA, including the microRNA fractions, was isolated by using animal tissue RNA purification kit purchased from Norgen (Canada). The concentration and quality of the RNAs were analyzed by NanoDrop spectrophotometer (Thermo Scientific, Wilmington, DE, USA). MicroRNA arrays were performed by a service provider (LC Sciences) using an array containing probes to 1900 mouse microRNA probes. Briefly, RNA samples were isolated, size fractionated, and labeled with Cy3 or Cy5. Samples were hybridized to dual-channel microarray using the μParaflo microfluidics chips of LC Sciences (Houston, TX). This array contained probes to mouse microRNAs listed in Sanger miRBase Release 21.0.

### RNA isolation and mRNA microarray

The same total RNA samples used in microRNA array was analyzed in Emory Integrated Genomics Core (EIGC) (Atlanta, GA) for mRNA expression microarray. Briefly, for quality control, all samples were analyzed using the Agilent 2100 Bioanalyzer system (agarose gel). The microarray experiment was performed using the Illumina iScan device which uses fluorescence detection of biotin-labelled cRNA. For each sample, 250 ng of total RNA was reverse transcribed, amplified and labeled with biotin-UTP using an Illumina. Total Prep Amp (RNA Amplification Kit (version 4393543, Ambion/Applied Biosystems). The quantity of labeled cRNA was measured using the NanoDrop spectrophotometer (Thermo Scientific), whereas the quality and size distribution of the labeled cRNA was assessed using the 2100 Bioanalyzer (Agilent). Finally, 1.5 mg biotin-labelled cRNA was hybridized to Illumina Mouse WG-6 v3 Expression Bead Chips according to the manufacturer’s protocol.

### HEK 293 cell culture and CsA treatment

The Human Embryonic Kidney (HEK) 293 (CRL-1573) cell line was purchased from the American Type Culture Collection (ATCC: Manassas, USA). Cells were cultured in Dulbecco's modified Eagle's medium (DMEM) supplemented with 10% fetal calf serum and antibiotics (100 U/ml penicillin and 100 μg/ml streptomycin) at 37°C in humidified atmosphere of 5% CO_2_. For the CsA treatment, HEK293 cells were seeded in 6-well plates with a density of 0.5 ×10^6^ cell/cm^2^. After reaching 80 to 90 percent of confluence, the cells was treated with CsA in the regular culture medium at concentrations of 1, 5, and 10 μg/ml and then incubated at 37°C in humidified atmosphere of 5% CO_2_. Total RNA was isolated from HEK293 cells using the mirVana miRNA Isolation Kit from Invitrogen (Waltham, Massachusetts). The manufacture’s protocol was followed. The concentration of products was measured by NanoDrop 2000 spectrophotometer.

### Statistical and bioinformatics analyses

MicroRNA expression data were pre-processed by LC Sciences and imported into the statistical software R for downstream analysis. The limma method was used to detect differentially expressed microRNAs [27]. The p-values were transformed to False Discovery Rates (FDRs) to address the issue of multiple testing [28]. We combined the FDR criterion (≤0.1) with fold change (≥2) and average signal strength (≥50) to select a list of differentially expressed micriRNAs.

Messenger RNA expression data was pre-processed by Illumina GenomeStudio software to obtain gene-level expression data. The data was then imported into the statistical software R for downstream analysis. We used the limma package for the detection of differentially expressed transcripts [27]. Given the distribution of fold change is different from that of the microRNA data, we used the criterion of FDR≤0.1 and fold change ≥1.25 to select the list of differentially expressed mRNAs.

### Gene ontology and pathway enrichment analysis

DIANA-miRPath v 2.0 pathway enrichment analysis (Papadopoulos et al., 2009) was used to detect molecular pathways that are targeted by the microRNAs that are differentially expressed between the control and the treatment groups. This application offers a series of tools specifically focused on microRNAs and target genes to all known KEGG pathways. Pathways showing a P value of ≤ 0.01 were considered significantly enriched. The DIANA-miRPath v2.0 can be accessed at the following address: http://diana.imis.athena-innovation.gr/DianaTools/index.php?r=mirpath/index.

### Quantitative Real–Time Polymerase Chain Reaction (qRT-PCR)

Quantitative RT-PCR was performed following the protocol of TaqMan MicroRNA Assays (Applied Biosystems, USA). cDNAs were generated using Taqman reverse transcription kit and TaqMan Universal PCR master mix according to manufacturer’s instructions. Resulting cDNA were used for quantitative real-time PCR using TaqMan mRNA assay (Applied Biosystems). Amplification and detection were performed on optical-grade 96-well plates using an iQ5 Cycler Multicolor real-time PCR detection system (Bio-Rad Laboratories, Hercules, CA, USA). The expression levels of mRNA were normalized to endogenous control miR-202. The relative microRNA level was calculated by the 2-ΔΔCt method and expressed as fold about vehicle treatment[27].

## Results

### CsA treatment induced the early sign of nephrotoxicity in mice kidney tissues

**Fig 1:**
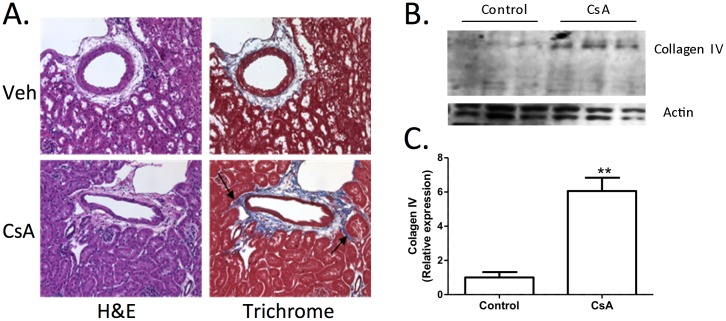
Mouse model of cyclosporine A induced nephrotoxicity. (A) Formalin-fixed kidney sections from CsA and vehicle-treated mice were thin-sectioned and then adjacent sections were stained with H&E or Trichrome. Peri-arteriolar regions were identified and representative images are shown. Arrows indicate expansion of cells that express collagen. B) Total protein lysates were collected from contralateral kidneys and expression of collagen IV was determined by western blotting. Each lane represents a different animal. C) Densitometric analysis of the proteins (*n* = 6) for each group segregated by CsA treatment and control. Error bars indicate s.e.m. *P<0.05 (two-tailed *t*-test).

Adult male C57BL/6 mice, a commonly used model of nephrotoxicity [28], were treated with CsA (CsA treatment group) or vehicle alone (control group) for six weeks. Kidney histology confirmed that CsA treatment induced cellular infiltration in the peri-arterial region (indicated with arrows) that coincides with an increased collagen expression that extends into the tubulointerstitial space (Fig 1A). Also, Western blotting of total protein lysates from the contralateral kidney confirmed increased expression of collagen IV in the CsA-treated mice group compared to control group (Fig 1B). The tubulointerstitial fibrosis and upregulation of collagen IV are early hallmarks of CsA nephrotoxicity. Densitometric analysis of the collagen expression level in the CsA-treated mice and control mice were compared (Fig 1C). The average of collagen IV expression in the CsA-treated mice was significantly higher than the average of collagen IV expression in the control mice. The combined histological image and Western blotting results demonstrated that CsA treatment for six weeks induced fibrosis in kidney tissue which is a hallmark for nephrotoxicity.

### Changes in expression profile of microRNA after CsA treatment

**Fig 2:**
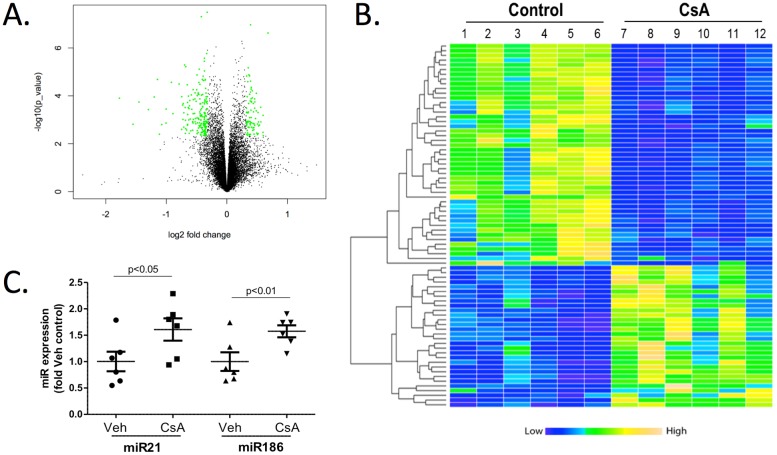
Changes in microRNA expression profile induced by CsA treatment in mice kidney tissue. A) The volcano plot of the microRNA array. Significantly altered microRNAs are indicated in green. B) The heatmap of selected differentially expressed microRNAs in control (lanes 1–6) and CsA-treated (lanes 7–12) mice. Increased expression is in yellow and decreased expression is in blue. C) Expression of miR-21 and miR-186 was confirmed by qRT-PCR in vehicle and CsA-treated total kidney mRNA.

To identify potential microRNAs and their roles in CsA-induced nephrotoxicity, we performed microRNA expression profiling in CsA-treated and control mice using μParaflo microfluidics chips of LC Sciences [29]. There are 1900 mouse microRNA probes on the chip. The microRNA array data were analyzed based on combined criteria of False Discovery Rate (≤0.1), fold change (≥2) and median signal strength (≥50). The volcano plot was shown in Fig 2A (the green dots indicate the differentially expressed microRNAs). Seventy-six (76) microRNAs were identified as significantly differentially expressed between the CsA treatment and control groups and are listed in Table 1. Among the 76 microRNAs, 30 microRNAs are significantly overexpressed with a fold change from 2.14 to 5, while expression of 47 microRNAs was suppressed with fold change from 0.13 to 0.49. The 76 differentially expressed miRs were converted into a heat map to show distinct microRNA expression profiling samples (Fig 2B). Each column represented a sample and each row showed one microRNA. Column one to six indicated the microRNA expression level of six control mice while lane seven to twelve indicated the six CsA-treated mice. Yellow represented overexpression of microRNAs while blue represented suppressed expression of microRNAs. These results demonstrated that CsA treatment substantially modified the microRNA expression profile in the mice kidney tissue. A concise table with fold changes and P values of the 76 differentially expressed microRNA was listed in Table A in S1 File.

**Table 1:** CsA-regulated microRNAs with ≥2.0-fold changes.

microRNA	Fold Change	FDR	Published role in the kidney	Ref
miR-19	5	0.025308		
miR-101	4.52	0.03254		
miR-29	4.12	0.045298	Renal fibrosis	
miR-106	3.44	0.020959		
miR-192	3.18	0.047575	Renal fibrosis,TGF beta	[17, 23, 35]
let-7	3.01	0.001637	Extracellular matrix (ECM) accumulation	[36]
miR-28	2.98	0.004771		
miR-203	2.92	0.044844		
miR-497a-5p	2.92	0.005035		
miR-28c	2.83	0.001561		
miR-199a-5p	2.8	0.005945		
mmu-miR-29c-3p	2.79	0.014215		
miR-140-3p	2.74	0.00815		
miR-130a-3p	2.73	0.037898	TGF beta	[16]
miR-322-5p	2.71	0.020008		
miR-148b-3p	2.59	0.043631		
miR-2137	2.56	0.046931		
miR-15a-5p	2.44	0.040385	TGF beta	[35]
miR-20b-5p	2.43	0.052909		
miR-151-5p	2.39	0.004771		
miR-350-3p	2.29	0.047575		
miR-186-5p	2.26	0.014246		
miR-200a-3p	2.23	0.001561	Renal fibrosis	[17]
miR-30e-3p	2.23	0.002595	TGF beta pathway	[23]
miR-21a-5p	2.19	0.021575	Renal fibrosis	[17]
miR-425-5p	2.17	0.013388		
miR-106a-5p	2.16	0.020959		
miR-322-3p	2.14	0.021151		
miR-6922-5p	0.49	0.051324		
miR-7212-3p	0.48	0.020959		
miR-3099-3p	0.46	0.02125		
miR-574-3p	0.45	0.00203		
miR-6896-3p	0.45	0.005035		
miR-467 cluster	0.43	0.00234		
miR-32-3p	0.41	0.005945		
miR-341-5p	0.41	0.01808		
miR-3082-5p	0.39	0.007958		
miR-574-5p	0.38	0.00246		
miR-1187	0.36	0.001561		
miR-568	0.36	0.013292		
miR-485-3p	0.35	0.004762		
miR-7027-3p	0.35	0.001465		
miR-5107-5p	0.34	0.026805		
miR-466 cluster	0.33	0.001561		[37]
miR-7082-5p	0.33	0.071538		
miR-6348	0.32	0.010492		
miR-7058-3p	0.31	0.000691		
miR-669 cluster	0.27	0.006272		
miR-7235-3p	0.25	0.00203		
miR-706	0.24	0.079161		
miR-6929-3p	0.21	0.001465		
miR-7056-5p	0.19	0.010194		
miR-709	0.17	0.001465		
miR-1195	0.13	0.001194		

To validate the microRNA expression data obtained from microRNA microarray results, the expression of miR-21 and miR-186 were compared between control group and CsA treatment group using the more sensitive qRT-PCR method. Fig 2C showed that both miR-21 and miR-186 were significantly overexpressed in the CsA-treated mice compared to control mice, and the trend was consistent with the microarray results.

### Changes in expression profile of mRNAafter CsA treatment

**Fig 3:**
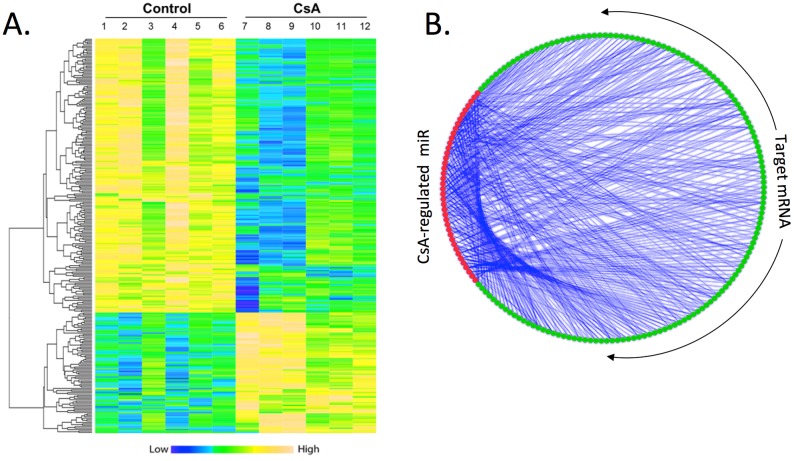
Changes in mRNA expression profile induced by CsA treatment in mice kidney tissue. A) The heatmap of selected differentially expressed mRNAs in control (lane 1–6) and CsA-treated (lanes 7–12) mice. Increased expression is in yellow and decreased expression is in blue. B) Regulatory relationship between the significant microRNAs and mRNAs. The red dots indicate the significantly regulated miRNA while the green dots indicate significantly regulated mRNA.

To study the genetic regulation in CsA-induced nephrotoxicity, the gene expression profiling was performed on the same mice kidney samples used in the microRNA array. The total RNAs were analyzed on Illumine Mouse WG-6 v2.0 Beadchips, with 33,678 transcripts. Six samples were studied simultaneously on a single BeadChip. Based on the array result, 278 genes were found to be significantly regulated. Compared to control mice, the expression of 85 genes were increased, while the expression of193 genes were suppressed in CsA treated mice. Fig 3 showed the heat map of the regulation of the top 278 regulated genes induced by CsA treatment (Fig 3A). Yellow indicated the over-expression of genes while blue indicated suppressed expression of genes. A concise table with fold changes and P values of the 278 differentially expressed genes was listed in Table B in S1 File.

### Identification of microRNA–gene network and pathways

Both the microRNA and mRNA array results revealed significant changes in expression pattern. To study the microRNA and gene regulating network, we used the microRNA package from Bioconductor [30, 31] to find the potential regulatory relationship between the significant differentially expressed microRNAs and mRNAs (Fig 3B). The red dots indicated the significantly changed expressed microRNAs while the green dots indicated significantly changed expressed mRNA. The microRNAs and their mRNA targets were linked with black straight lines.

To further understand the potential impact of aberrantly expressed microRNAs on biological processes related to CsA treatment, the pathway enrichment analysis was done using Diana miRPath, a web-based computational tool that allows a biological interpretation of microRNA profiling. Enrichment analysis was performed considering the 76 statistically significantly changed microRNAs, whose predicted targeted genes are mapped to different functional cellular pathways. The top 20 pathways of the regulated miRNAs were listed in Table 2. The KEGG pathway enrichment analysis revealed that the significantly changed microRNAs regulate the genes involved in pathways strongly relevant to nephrotoxicity, such as MAPK, PI3K-Akt signaling, Wnt, Erb, insulin signaling, TGF-β, mTOR, VEGF signaling pathway signaling pathway.

**Table 2:** The specific biological pathways regulated by differentially expressed microRNAs.

KEGG Pathway	P–value
**Pathways in cancer**	8.42E-76
**PI3K-Akt signaling pathway**	4.52E-67
**MAPK signaling pathway**	1.21E-52
**Endocytosis**	6.85E-45
**Focal adhesion**	1.96E-43
**Regulation of actin cytoskeleton**	1.20E-42
**Wnt signaling pathway**	1.02E-38
**Axon guidance**	4.07E-32
**Ubiquitin mediated proteolysis**	9.52E-28
**Insulin signaling pathway**	3.40E-26
**Neurotrophin signaling pathway**	5.46E-25
**T cell receptor signaling pathway**	3.90E-24
**Adherens junction**	3.95E-23
**Osteoclast differentiation**	3.95E-23
**Dopaminergic synapse**	1.49E-22
**ErbB signaling pathway**	5.82E-22
**HTLV-I infection**	5.82E-22
**Prostate cancer**	2.86E-21
**Melanogenesis**	8.90E-21
**Renal cell carcinoma**	8.90E-21
**TGF-beta signaling pathway**	6.70E-20

### *In vitro* study in HEK 293 cell line

**Fig 4:**
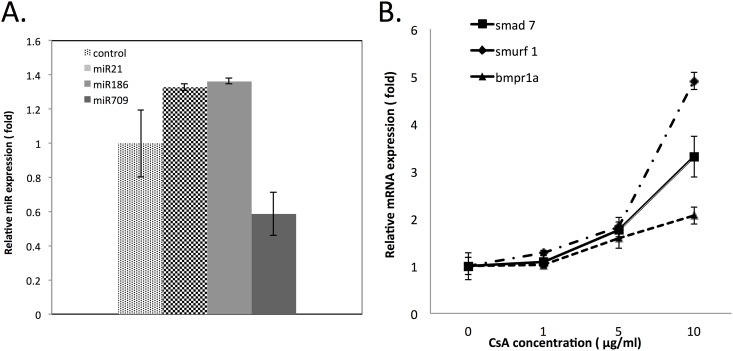
Validation of change of expression of selected microRNAs and mRNAs in the HEK 293 in vitro cell model. A) Expression of miR-21, miR-186 and miR-709 in response to CsA treatment at 1 μg/ml for 24 hours. The vehicle control for the experiment was complete media. Control for qRT-PCR experiment was snoRNA 202 (* indicate significant change, P≤0.05). B) Expression of mRNAs, BMPR1A, SMURF1, and SMAD7 in response to CsA treatment at concentrations of 1, 5, 10 μg/ml after 48 hours. Control for qRT-PCR was β actin. (* indicate significant change, P≤0.01).

To validate that the change of the expression of microRNAs and mRNAs in the mice model are directly related to CsA treatment, we used HEK 293 cell line as an *in vitro* model. Three microRNAs (miR-21, miR-186, and miR-709) and three mRNAs (SMAD7, SMURF1 and BMPR1a) were selected to validate whether the same trend of change will be induced by CsA treatment in HEK 293 cells. The expression level change of miR-21, miR-186 and miR-709 was shown in Fig 4A. Compared to the control, miR-21 expression level increased 1.33-fold, miR-186 expression level increased 1.36-fold, while miR-709 decreased to 0.58-fold. The results observed in the HEK 293 cells (miR-21 and miR-186 increased while miR-709 decrease), were similar to the results observed in the animal model. At the same time, the expression change of three mRNAs induced by CsA treatment was shown in Fig 4B. HEK 293 cells were treated with CsA at 0, 1, 5, 10 μg/ml. After 48 hours, the cells were collected and lysed for total RNA isolation. The expression of all three mRNAs, SMAD7, SMURF1, and BMPR1a, were significantly increased at 10 μg/ml concentration. BMPR1 was also significantly increased after treatment with CsA at 5 μg/ml after 48 hours. The expression level of the three selected mRNAs showed a dose-dependent response to CsA treatment.

## Discussion

The introduction of the calcineurin inhibitor cyclosporine in solid organ transplantation revolutionized transplantation medicine. The immunosuppressive activities result from inhibition of phosphatase activity of calcineurin, leading to the suppression of T cell activation. However, the inhibition of calcineurin is not T cell specific. The nephrotoxic effects of calcineurin inhibitors are the major side effects. Nephrotoxicity is a complicated physiological process involving complex regulation of gene expression and protein interaction. Although the exact mechanisms leading to renal dysfunction are not completely understood, this impairment is considered to be involved with the inflammatory response, TGFβ overexpression, oxidative stress, renin-angiotensin-aldosterone system (RAAS) activation, which combined to induce fibrosis and apoptosis [22, 23].

In this study, we systematically investigated parallel microRNA and mRNA expression profiling change related to CsA treatment in C57BL/6 mice model. Parallel mRNA and microRNA expression profiling by microarray revealed significant differentially expressed 268 genes and 76 microRNAs (The complete microRNA and mRNA list with P values are listed in Tables A and B in S1 File). The data indicated that CsA treatment substantially modified both the microRNA and mRNA expression. Certain changes in microRNA expression seen in our study were anticipated. These changes have been reported by previous studies with conventional research methods. Among the microRNAs whose expression were modified more than two-fold, there were 25 microRNAs that have not been reported to be related to either kidney fibrosis or CsA. For example, miR-466, miR-669, and miR-467 were derived from a single microRNA cluster Chromosome 2 microRNA cluster (C2MC) which might play important roles in immune response [32]. But other microRNAs, have not been reported to be related to either immune response or kidney nephrotoxicity. One example is miR-709 which is decreased to fold 0.17 in the CsA-treated mice. There is no report on the effect of miR-709 being related to nephrotoxicity, but miR-709 has been identified as the nuclear-enriched microRNA with the ability to regulate other microRNA biogenesis [33].

Next, we mapped the 76 selected microRNAs to putative target mRNA networks using DIANA-miRPath v 2.0. The pathway enrichment analysis gives a more systemic understanding of the mechanism. Of the top 20 pathways listed (Table 2), some of the pathways are related to the immune inhibition functions, such as T cell receptor signaling pathway. At the same time, there are many pathways that have been suggested to be related to the adverse side effects of CsA. For example, TGFβ signaling pathway plays a key role in CsA-induced fibrosis. Among the 76 differentially expressed microRNAs, 16 microRNA/mRNA clusters were identified that regulate genes that are involved in the TGFβ signaling pathway. Also, we searched the literature for publications that linked the remaining differentially expressed microRNAs to TGFβ expression or signaling or fibrosis in the kidney. The 20 microRNAs that are linked to TGFβ expression or signaling or fibrosis in the kidney are listed in (Table 3). MicroRNAs in red are those that have been previously linked to TGFβ and fibrosis in the kidney. Those in blue have gene targets predicted to be involved in TGFβ or fibrosis but with no published reports linking them to fibrosis or TGFβ in the kidney. Significantly, only miR-21 has previously been linked to CsA.

**Table 3:** Differentially expressed microRNAs in CsA treated mice that have predicted or published target mRNAs related to TGFβ and/or renal fibrosis. MicroRNAs in red are those that have been previously linked to TGFβ and fibrosis in the kidney. Those in blue have gene targets predicted to be involved in TGFβ or fibrosis but with no published reports linking them to fibrosis or TGFβ in the kidney.

miRNA	Fold Δ	Predicted or published gene targets in TGFβ pathway	Refs
**miR-709**	0.17	Ppp2r1b, Thbs1, Skp1a, Myc, Smad5,	
**miR-1187**	0.36	Rock2, Smad4, Inhbb, Bmp7, Smad1, Bmp4	
**miR-32**	0.41	Ppp2r1b, Rock1, Thbs2, Ppp2cb, Acvr1, Acvr1c	
**miR-669 cluster**	0.44	Acvr1, Smad1, Pitx2, Id2, Smad7, Acvr1c	
**miR-466/467 cluster**	0.46	Smad6, Smad9, Pitx2, Bmpr1b, Ppp2ca, Thbs2, Bmp5, Id3, Nodal, Fst, Rbl1	
**miR-322 3p,5p**	2.14	Mapk1, Pitx2, Rock1, Bmpr1b, Fst, Acvr1c	
**miR-106a**	2.16	Smad6, E2f5, Zfyve9, Tgfbr2, Rock2, Smad7, Rbl1	
**miR-21a**	2.19	Acvr2a, Smad7, PAI-1, col1, col2a, fibronectin, MMP9, TIMP1	
**miR-30e**	2.23	cTGF, UCP2, Ecad, FH, αSMA	
**miR-200a**	2.23	FOG2, TGFβ2, fibronectin	[25, 26]
**miR-186-5p**	2.26	Smad6, Tgfb2, Mapk1, Bmpr1a, Gdf6, Nog, Ppp2ca, Tgfbr2, Smurf2, Sp1,	
**miR-350-3p**	2.29	E2f5, Rock1, Rhoa, Acvr2a, Smad5	
**miR-20b**	2.43	Smad6, E2f5, Zfyve9, Tgfbr2, Rock2, Smad7, Rbl1	
**miR-15a**	2.44	Ppp2r1b, Smurf1, Acvr2a, Smurf2, Smad7, Smad5	[35, 38]
**miR-322-5p**	2.71	Ppp2r1b, Smurf1, Acvr2a, Mapk3, Smad7, Smad5	
**miR-130a**	2.73	Bmpr1a, Ppp2r1b, Smurf1, Acvr2a, Smurf2, Smad7, Smad5	[16]
**miR-29c**	2.79	colI, colIV, Spry1	[22]
**miR-28a,c**	2.83	Ppp2r1b, Ppp2r1a	
**Let-7d**	3.01	colI, colIV, TGFβR1	[20]
**miR-192-3p**	3.18	Zeb1/2, SIP1, Ecad, αSMA	[24]

We further analyzed mRNA expression in the same kidney samples. Results were analyzed compared to mRNA functional modules as defined by Gene Ontology (GO) consortium [34]. A potential regulatory relationship between the significant differentially expressed microRNAs and mRNAs was observed (Fig 3B). In particular, BMP5 mRNA is upregulated while the miR-466/467 cluster is downregulated. SP1 mRNA is downregulated while miR-186 is increased after CsA treatment. To validate the data of microarray, the expression of candidate microRNAs and mRNA were validated using more sensitive quantification method qRT-PCR. We selected miR-21 because it has previously been reported to be regulated after CsA treatment. We also selected miR-186; whose predicted gene targets involve in kidney fibrosis such as bone morphogenetic protein 7 (BMP7), transcription factor SP1, SMAD family member 1 (SMAD1), and inhibitor of DNA binding 4 (ID4).

To further verify the observed expression change of microRNA and mRNA in the mice is directly related CsA treatment, HEK 293 cell line was used as an *in vitro* model to test the CsA treatment induced microRNA and mRNA expression change. MiR-21, miR-186 and miR-709 were selected as the candidate microRNAs to test whether parallel changes in expression level can also be induced in the *in vitro* cell model. Two widely investigated miRNAs, miR-21 and miR-709, were chosen. MiR-21 represented the increased expressed microRNAs (increased 2.19 fold in kidney tissue). While miR-709 represented the decreased expressed of microRNAs (decreased to 0.168 fold in kidney tissue). At the same time, miR-186 (increased 2.26 fold in kidney tissue) was chosen as a miRNA that has not been widely investigated. Three mRNAs were also selected to investigate the mRNA expression changes related to CsA treatment in HEK 293 cell. BMPR1A (bone morphogenic protein receptor type 1 A), SMAD7 (SMAD Family Member 7) and SMURF1 (SMAD specific E3 ubiquitin protein ligase 1) are the predicted regulation targets of miR-86, miR 21 and many other miRs. Also, BMPR1A, SMAD7, SMURF1 have been reported to be related to kidney fibrosis which is one key indicator of kidney nephrotoxicity. The expression of selected microRNAs and genes showed same trend of change in the *in vitro* cell model after CsAtreatment compared to the change observed in the CsA treated mice kidney tissue.

Our research contributes to the understanding of the role of microRNAs in CsA-induced kidney nephrotoxicity in a systematic way. Furthermore, the knowledge of microRNA regulatory pathway may help us discover the microRNA molecule or combination of microRNAs that have therapeutic potential in preventing and/or reversing kidney nephrotoxicity associated with CsA treatment.

## Conclusion

Our study profiled expression of microRNA and mRNA genes in mouse kidney after kidney fibrosis has been induced (the major sign of kidney toxicity) and pairwisely compared the expression profiles. The results showed that cyclosporine A caused intensive change in both microRNA and gene expression compared to control mice. In addition, the microRNA and mRNA profiling results showed close correlation. Among the differentially expressed miRNAs, we observed the miR-21, miR-192, miR-29, miR-30 which have been associated with the kidney fibrosis by several independent studies. Our results are in agreement with published results. More interestingly, our results discovered some novel miRNAs that have not been reported to be involved in kidney or CsA, such as miR-186, miR-709, and so on.

## Supporting information

S1 File Supporting tables of differentially expressed miRNA and mRNA. (DOCX)
