# Supplementary material for: Cyclosporine A alters expression of renal microRNAs: New insights into calcineurin inhibitor nephrotoxicity
Source: PLoS One. 2017 Apr 17;12(4):e0175242. doi: 10.1371/journal.pone.0175242 (PMC5393575; doi:10.1371/journal.pone.0175242)
Supplement: S1 File — (DOCX) [file pone.0175242.s001.docx]

Supplementary File

Table A. List of selected micro RNAs.

| Name | FDR | log2 fold change |
| --- | --- | --- |
| mmu-miR-1195 | 0.001194449 | -2.900549069 |
| mmu-miR-709 | 0.001464763 | -2.571803431 |
| mmu-miR-7056-5p | 0.010193819 | -2.384859351 |
| mmu-miR-6929-3p | 0.001464763 | -2.273672926 |
| mmu-miR-706 | 0.079160962 | -2.037754922 |
| mmu-miR-7235-3p | 0.00202988 | -2.00757322 |
| mmu-miR-669h-3p | 0.006271629 | -1.890928301 |
| mmu-miR-7058-3p | 0.000690784 | -1.674904626 |
| mmu-miR-6348 | 0.010492408 | -1.636764931 |
| mmu-miR-7082-5p | 0.071537746 | -1.595554204 |
| mmu-miR-466i-5p | 0.001561231 | -1.587266436 |
| mmu-miR-466d-3p | 0.00552595 | -1.568347787 |
| mmu-miR-5107-5p | 0.02680545 | -1.53556668 |
| mmu-miR-7027-3p | 0.001464763 | -1.533069516 |
| mmu-miR-485-3p | 0.004762357 | -1.528320503 |
| mmu-miR-568 | 0.013291893 | -1.49102229 |
| mmu-miR-1187 | 0.001561231 | -1.471489862 |
| mmu-miR-669n | 0.007983074 | -1.444967705 |
| mmu-miR-669c-5p | 0.007836512 | -1.39238876 |
| mmu-miR-669o-5p | 0.010193819 | -1.381283373 |
| mmu-miR-574-5p | 0.002459689 | -1.380100995 |
| mmu-miR-3082-5p | 0.007957686 | -1.374306388 |
| mmu-miR-466m-5p | 0.008150454 | -1.3584283 |
| mmu-miR-466g | 0.001774555 | -1.302775824 |
| mmu-miR-341-5p | 0.018080264 | -1.28438686 |
| mmu-miR-466i-3p | 0.001464763 | -1.276140846 |
| mmu-miR-32-3p | 0.005945271 | -1.27321989 |
| mmu-miR-466h-3p | 0.001464763 | -1.272571227 |
| mmu-miR-669c-3p | 0.004476769 | -1.231083382 |
| mmu-miR-467f | 0.002339728 | -1.216807478 |
| mmu-miR-466q | 0.001818984 | -1.215117549 |
| mmu-miR-466f-3p | 0.001464763 | -1.209829301 |
| mmu-miR-669l-5p | 0.019827217 | -1.192645078 |
| mmu-miR-669p-3p | 0.001876098 | -1.187857898 |
| mmu-miR-669f-5p | 0.008150454 | -1.180794387 |
| mmu-miR-6896-3p | 0.005034787 | -1.163680787 |
| mmu-miR-574-3p | 0.00202988 | -1.162058182 |
| mmu-miR-466m-3p | 0.006218409 | -1.150993032 |
| mmu-miR-467a-3p | 0.006685485 | -1.135684677 |
| mmu-miR-3099-3p | 0.021250189 | -1.118072994 |
| mmu-miR-669e-3p | 0.009731034 | -1.115605806 |
| mmu-miR-467d-3p | 0.006528924 | -1.106625795 |
| mmu-miR-467b-3p | 0.005018997 | -1.096899894 |
| mmu-miR-669f-3p | 0.005018997 | -1.096861539 |
| mmu-miR-467e-3p | 0.012936176 | -1.071358311 |
| mmu-miR-7212-3p | 0.020958619 | -1.057678276 |
| mmu-miR-6922-5p | 0.051324065 | -1.014597376 |
| mmu-miR-322-3p | 0.021150908 | 1.099929799 |
| mmu-miR-106a-5p | 0.020958619 | 1.108326153 |
| mmu-miR-425-5p | 0.013388107 | 1.114559764 |
| mmu-miR-21a-5p | 0.021574898 | 1.12982811 |
| mmu-miR-30e-3p | 0.002594844 | 1.158514017 |
| mmu-miR-200a-3p | 0.001561231 | 1.159926449 |
| mmu-miR-186-5p | 0.014246223 | 1.177787119 |
| mmu-miR-350-3p | 0.04757524 | 1.195550809 |
| mmu-miR-151-5p | 0.004771224 | 1.256254919 |
| mmu-miR-20b-5p | 0.05290884 | 1.283440616 |
| mmu-miR-15a-5p | 0.040384712 | 1.28397801 |
| mmu-miR-2137 | 0.046931455 | 1.354474775 |
| mmu-miR-148b-3p | 0.043630503 | 1.373565639 |
| mmu-miR-322-5p | 0.020008004 | 1.438985309 |
| mmu-miR-130a-3p | 0.037897606 | 1.449475323 |
| mmu-miR-140-3p | 0.008150454 | 1.453102385 |
| mmu-miR-29c-3p | 0.01421471 | 1.478755107 |
| mmu-miR-199a-5p | 0.005945271 | 1.485426827 |
| mmu-miR-28c | 0.001561231 | 1.498837746 |
| mmu-miR-497a-5p | 0.005034787 | 1.543987523 |
| mmu-miR-203-3p | 0.044844197 | 1.546129855 |
| mmu-miR-28a-5p | 0.004771224 | 1.575860294 |
| mmu-let-7d-3p | 0.001636602 | 1.589032165 |
| mmu-miR-192-3p | 0.04757524 | 1.670876404 |
| mmu-miR-106b-5p | 0.020958619 | 1.781088147 |
| mmu-miR-29b-3p | 0.045297609 | 2.043928578 |
| mmu-miR-101c | 0.025301076 | 2.145197916 |
| mmu-miR-101a-3p | 0.032540051 | 2.177712848 |
| mmu-miR-19b-3p | 0.025307918 | 2.322600523 |

Table B. List of selected mRNAs.

| ENTREZ_ID | Gene_Name | BH FDR | log2 Fold Change |
| --- | --- | --- | --- |
| 319317 | small nucleolar RNA host gene 11 | 0.019798191 | -1.774129536 |
| 100041388 | predicted gene 3308 | 0.057289777 | -1.549622922 |
| 66961 | nuclear paraspeckle assembly transcript 1 (non-protein coding) | 0.022364842 | -1.45045712 |
| 72373 | prostate stem cell antigen | 0.031996854 | -1.295982582 |
| 77866 | RIKEN cDNA E130102H24 gene | 0.018248305 | -1.199558809 |
| 14677 | guanine nucleotide binding protein (G protein), alpha inhibiting 1 | 0.009483162 | -1.14655779 |
| 170942 | erythroid differentiation regulator 1 | 0.091607496 | -1.114038973 |
| 58805 | MLX interacting protein-like | 0.058075329 | -1.074625884 |
| 15412 | homeobox B4 | 0.054646087 | -1.00465043 |
| 236604 | phosphatidylserine decarboxylase, pseudogene 1 | 0.031350528 | -1.002427619 |
| 66625 | PNN interacting serine/arginine-rich | 0.010357797 | -0.915856985 |
| 72522 | ataxin 7-like 2 | 0.037805729 | -0.890316336 |
| 28027 | 28027 | 0.010806106 | -0.788174068 |
| 100040243 | 100040243 | 0.072073042 | -0.746135985 |
| 227648 | SEC16 homolog A (S. cerevisiae) | 0.018196193 | -0.744718314 |
| 70772 | gametogenetin binding protein 1 | 0.025763645 | -0.729845578 |
| 15436 | homeobox D4 | 0.048618736 | -0.7257048 |
| 68490 | zinc finger protein 579 | 0.023675578 | -0.719940064 |
| 232811 | suppressor of variegation 4-20 homolog 2 (Drosophila) | 0.064605437 | -0.710898808 |
| 396184 | fibronectin leucine rich transmembrane protein 1 | 0.03692118 | -0.710688022 |
| 74200 | RIKEN cDNA 2810403A07 gene | 0.00537421 | -0.697099941 |
| 212483 | family with sequence similarity 193, member B | 0.028810846 | -0.688408085 |
| 216976 | cDNA sequence BC030499 | 0.08747694 | -0.685317024 |
| 319517 | 319517 | 0.05004555 | -0.683805858 |
| 15441 | heterochromatin protein 1, binding protein 3 | 0.068352182 | -0.679231953 |
| 230249 | expressed sequence AI314180 | 0.005779452 | -0.677740993 |
| 56868 | pregnancy-specific glycoprotein 23 | 0.074044437 | -0.663859538 |
| 100047264 | 100047264 | 0.010807222 | -0.654226609 |
| 320790 | chromodomain helicase DNA binding protein 7 | 0.059039923 | -0.650388613 |
| 100041567 | NSFL1 (p97) cofactor (p47) pseudogene | 0.022640434 | -0.649425195 |
| 207686 | cilia and flagella associated protein 69 | 0.057458258 | -0.647951332 |
| 20495 | solute carrier family 12, member 1 | 0.038824771 | -0.646551698 |
| 56312 | nuclear protein transcription regulator 1 | 0.028810846 | -0.637545461 |
| 74493 | tankyrase, TRF1-interacting ankyrin-related ADP-ribose polymerase 2 | 0.018196193 | -0.634266327 |
| 20384 | serine/arginine-rich splicing factor 5 | 0.049386458 | -0.628918923 |
| 231807 | cDNA sequence BC037034 | 0.030461409 | -0.625554785 |
| 100045005 | 100045005 | 0.08747694 | -0.621156044 |
| 53319 | nuclear RNA export factor 1 | 0.026431964 | -0.618413456 |
| 73095 | solute carrier family 25, member 42 | 0.021585686 | -0.611988703 |
| 79263 | tripartite motif-containing 39 | 0.04770923 | -0.604428472 |
| 15258 | homeodomain interacting protein kinase 2 | 0.076687042 | -0.595143598 |
| 320319 | 320319 | 0.016885797 | -0.587214604 |
| 434168 | predicted gene 5590 | 0.031414489 | -0.579187914 |
| 380950 | 380950 | 0.074389989 | -0.563712116 |
| 72102 | dual specificity phosphatase 11 (RNA/RNP complex 1-interacting) | 0.053827365 | -0.563393262 |
| 27399 | inositol hexaphosphate kinase 1 | 0.052656515 | -0.563023495 |
| 100038635 | predicted gene 10621 | 0.054644073 | -0.558303503 |
| 106952 | ArfGAP with RhoGAP domain, ankyrin repeat and PH domain 3 | 0.037805729 | -0.557639483 |
| 67684 | LUC7-like 3 (S. cerevisiae) | 0.01399909 | -0.556807315 |
| 214469 | family with sequence similarity 168, member B | 0.020882724 | -0.554320034 |
| 235493 | family with sequence similarity 214, member A | 0.048618736 | -0.553518346 |
| 215445 | RAB11 family interacting protein 3 (class II) | 0.037805729 | -0.532566987 |
| 72699 | Lck interacting transmembrane adaptor 1 | 0.02003218 | -0.527027713 |
| 15434 | homeobox D3 | 0.016275807 | -0.524546192 |
| 13859 | epidermal growth factor receptor pathway substrate 15-like 1 | 0.052826034 | -0.523631547 |
| 19732 | ral guanine nucleotide dissociation stimulator-like 2 | 0.046566126 | -0.519555332 |
| 12750 | CDC like kinase 4 | 0.055981649 | -0.512780521 |
| 16581 | kinesin family member C2 | 0.021754619 | -0.50760625 |
| 100040085 | predicted gene 2589 | 0.01399909 | -0.497796552 |
| 245007 | zinc finger and BTB domain containing 38 | 0.008901945 | -0.493699713 |
| 231003 | kelch-like 17 | 0.045385297 | -0.49236193 |
| 56706 | cyclin L1 | 0.088981251 | -0.490867613 |
| 432554 | DEAD (Asp-Glu-Ala-Asp) box polypeptide 5 pseudogene | 0.017668135 | -0.489588401 |
| 230872 | ciliary rootlet coiled-coil, rootletin | 0.075767897 | -0.487521591 |
| 384309 | tripartite motif-containing 56 | 0.054570083 | -0.484189889 |
| 106205 | zinc finger CCCH type containing 7 A | 0.016275807 | -0.474218819 |
| 59013 | heterogeneous nuclear ribonucleoprotein H1 | 0.083720982 | -0.473102429 |
| 108155 | O-linked N-acetylglucosamine (GlcNAc) transferase (UDP-N-acetylglucosamine:polypeptide-N-acetylglucosaminyl transferase) | 0.016616843 | -0.472588808 |
| 20497 | solute carrier family 12, member 3 | 0.046218154 | -0.469351164 |
| 81879 | transcription factor CP2-like 1 | 0.065891256 | -0.468994758 |
| 225929 | protein associated with topoisomerase II homolog 1 (yeast) | 0.048618736 | -0.463825841 |
| 79566 | SH3 binding domain protein 5 like | 0.010357797 | -0.462304072 |
| 75410 | lysine (K)-specific methyltransferase 2B | 0.016616843 | -0.46227649 |
| 231326 | aminoadipate-semialdehyde dehydrogenase | 0.06016288 | -0.457716146 |
| 110809 | serine/arginine-rich splicing factor 1 | 0.006723454 | -0.456606963 |
| 68671 | phosphate cytidylyltransferase 2, ethanolamine | 0.060892046 | -0.454000553 |
| 72722 | family with sequence similarity 98, member A | 0.054570083 | -0.453201546 |
| 545428 | coiled-coil domain containing 141 | 0.035774202 | -0.44718055 |
| 20403 | intersectin 2 | 0.02003218 | -0.445432588 |
| 239170 | family with sequence similarity 160, member B2 | 0.010806106 | -0.445187413 |
| 102334 | ankyrin repeat domain 10 | 0.064605437 | -0.444866724 |
| 67213 | CKLF-like MARVEL transmembrane domain containing 6 | 0.023675578 | -0.442694035 |
| 234373 | SURP and G patch domain containing 2 | 0.037805729 | -0.44166018 |
| 103135 | PAN2 polyA specific ribonuclease subunit homolog (S. cerevisiae) | 0.040833969 | -0.439765464 |
| 53817 | DEAD (Asp-Glu-Ala-Asp) box polypeptide 39B | 0.049386458 | -0.434467022 |
| 22339 | vascular endothelial growth factor A | 0.09343867 | -0.43280534 |
| 19017 | peroxisome proliferative activated receptor, gamma, coactivator 1 alpha | 0.032665295 | -0.43246956 |
| 110524 | diacylglycerol kinase, theta | 0.023675578 | -0.431410623 |
| 217684 | sushi domain containing 6 | 0.059564802 | -0.431218691 |
| 99412 | golgi autoantigen, golgin subfamily a, 2 | 0.05655675 | -0.428144928 |
| 100047834 | 100047834 | 0.040833969 | -0.427545087 |
| 105246 | bromodomain containing 9 | 0.09607484 | -0.427218364 |
| 16776 | laminin, alpha 5 | 0.049386458 | -0.426341667 |
| 12509 | CD59a antigen | 0.042060214 | -0.424869906 |
| 319601 | zinc finger protein 653 | 0.084571728 | -0.424534647 |
| 240215 | solute carrier family 4, sodium bicarbonate cotransporter, member 9 | 0.039844977 | -0.424376237 |
| 106064 | expressed sequence AW549877 | 0.00051341 | -0.421957563 |
| 20637 | small nuclear ribonucleoprotein 70 (U1) | 0.065126671 | -0.420394768 |
| 103784 | WD repeat domain 92 | 0.055593086 | -0.419198829 |
| 228889 | DEAD (Asp-Glu-Ala-Asp) box polypeptide 27 | 0.017167685 | -0.418123007 |
| 67040 | DEAD (Asp-Glu-Ala-Asp) box polypeptide 17 | 0.030313851 | -0.417604451 |
| 320590 | SV2 related protein homolog (rat)-like | 0.039289727 | -0.415834495 |
| 231876 | lemur tyrosine kinase 2 | 0.066322983 | -0.415552493 |
| 100045887 | 100045887 | 0.039871959 | -0.415548613 |
| 21687 | endothelial-specific receptor tyrosine kinase | 0.082093943 | -0.415543875 |
| 20481 | ski sarcoma viral oncogene homolog (avian) | 0.083100422 | -0.412743312 |
| 21886 | transducin-like enhancer of split 2, homolog of Drosophila E(spl) | 0.083100422 | -0.411116694 |
| 243725 | protein phosphatase 1, regulatory (inhibitor) subunit 9A | 0.065838242 | -0.410184803 |
| 67302 | zinc finger CCCH type containing 13 | 0.009483162 | -0.404376327 |
| 12385 | catenin (cadherin associated protein), alpha 1 | 0.072643268 | -0.403155003 |
| 625098 | solute carrier family 38, member 6 | 0.065360235 | -0.401678522 |
| 353156 | EGF-like domain 7 | 0.027787744 | -0.400737515 |
| 100045983 | 100045983 | 0.016616843 | -0.399491699 |
| 54446 | nuclear factor of activated T cells 5 | 0.02811231 | -0.398379635 |
| 226976 | KAT8 regulatory NSL complex subunit 3 | 0.022852136 | -0.397426709 |
| 19156 | prosaposin | 0.081070758 | -0.396622806 |
| 236920 | START domain containing 8 | 0.042695998 | -0.396175677 |
| 74315 | ring finger protein 145 | 0.008901945 | -0.39170217 |
| 319740 | zinc finger, FYVE domain containing 27 | 0.077103633 | -0.389107623 |
| 27223 | transformation related protein 53 binding protein 1 | 0.009560263 | -0.388691732 |
| 14388 | growth factor receptor bound protein 2-associated protein 1 | 0.09751693 | -0.388228379 |
| 71752 | general transcription factor IIIC, polypeptide 2, beta | 0.058075329 | -0.387176707 |
| 12974 | citrate synthase | 0.054662046 | -0.386353672 |
| 209456 | transformation related protein 53 binding protein 2 | 0.059702462 | -0.380700806 |
| 18597 | pyruvate dehydrogenase E1 alpha 1 | 0.052120744 | -0.380605528 |
| 77036 | RIKEN cDNA 1700109H08 gene | 0.057458258 | -0.376098068 |
| 100044468 | 100044468 | 0.088981251 | -0.373711719 |
| 319939 | tensin 3 | 0.048618736 | -0.373536969 |
| 216190 | adaptor protein, phosphotyrosine interaction, PH domain and leucine zipper containing 2 | 0.036631652 | -0.373370401 |
| 56480 | TANK-binding kinase 1 | 0.004480954 | -0.372612383 |
| 20924 | suppressor of Ty 5 | 0.052826034 | -0.37223337 |
| 103836 | zinc finger protein 692 | 0.045005329 | -0.371586213 |
| 100273 | oxysterol binding protein-like 9 | 0.004023414 | -0.371546476 |
| 17756 | microtubule-associated protein 2 | 0.088755559 | -0.371444027 |
| 53861 | zinc finger, RAN-binding domain containing 2 | 0.037196195 | -0.370440788 |
| 22710 | zinc finger protein 52 | 0.080583751 | -0.37042626 |
| 78920 | dihydrolipoamide S-succinyltransferase (E2 component of 2-oxo-glutarate complex) | 0.023675578 | -0.370328012 |
| 217039 | gametogenetin binding protein 2 | 0.007063719 | -0.369954563 |
| 77832 | trichoplein, keratin filament binding | 0.074617699 | -0.368733282 |
| 226744 | consortin, connexin sorting protein | 0.041664973 | -0.366738402 |
| 100048330 | 100048330 | 0.022364842 | -0.366434214 |
| 94353 | high mobility group nucleosomal binding domain 3 | 0.08747694 | -0.366330056 |
| 104367 | small nucleolar RNA, H/ACA box 65 | 0.063306107 | -0.365930423 |
| 70650 | zinc finger, CCHC domain containing 8 | 0.08747694 | -0.365581326 |
| 94091 | tripartite motif-containing 11 | 0.020976656 | -0.365364766 |
| 12400 | core binding factor beta | 0.063162195 | -0.364598897 |
| 319322 | splicing factor 3b, subunit 2 | 0.094203887 | -0.364551416 |
| 277010 | MARVEL (membrane-associating) domain containing 1 | 0.037944651 | -0.363854237 |
| 54353 | src family associated phosphoprotein 2 | 0.055515757 | -0.363684827 |
| 11931 | ATPase, Na+/K+ transporting, beta 1 polypeptide | 0.037001381 | -0.363431312 |
| 20382 | serine/arginine-rich splicing factor 2 | 0.072643268 | -0.361920896 |
| 28071 | TWIST neighbor | 0.064882443 | -0.361315872 |
| 17257 | methyl CpG binding protein 2 | 0.009560263 | -0.360246994 |
| 545725 | mitochondrial transcription termination factor 1a | 0.061649809 | -0.357979876 |
| 234684 | leucine rich repeat containing 29 | 0.058075329 | -0.357466358 |
| 66711 | Shwachman-Bodian-Diamond syndrome homolog (human) | 0.021585686 | -0.356907288 |
| 235582 | glycerate kinase | 0.086625488 | -0.356850629 |
| 21844 | T cell lymphoma invasion and metastasis 1 | 0.037547976 | -0.355784311 |
| 11938 | ATPase, Ca++ transporting, cardiac muscle, slow twitch 2 | 0.009483162 | -0.355130143 |
| 53330 | vesicle-associated membrane protein 4 | 0.058075329 | -0.354367523 |
| 74477 | RIKEN cDNA 4933427D14 gene | 0.051904298 | -0.352881667 |
| 22344 | vascular endothelial zinc finger 1 | 0.012864806 | -0.350432803 |
| 68108 | RIKEN cDNA 9430008C03 gene | 0.090376545 | -0.349391963 |
| 434077 | BRO1 domain and CAAX motif containing pseudogene | 0.07809172 | -0.349239981 |
| 110593 | PR domain containing 2, with ZNF domain | 0.040426592 | -0.348110349 |
| 170822 | ubiquitin specific peptidase 33 | 0.068809662 | -0.347681761 |
| 76936 | heterogeneous nuclear ribonucleoprotein M | 0.016313056 | -0.345044786 |
| 11699 | alpha 1 microglobulin/bikunin | 0.065750331 | -0.344986011 |
| 213109 | PHD finger protein 3 | 0.099520769 | -0.343778587 |
| 105348 | golgi membrane protein 1 | 0.064605437 | -0.343030769 |
| 72503 | RIKEN cDNA 2610507B11 gene | 0.098217646 | -0.342464461 |
| 225791 | zinc binding alcohol dehydrogenase, domain containing 2 | 0.018196193 | -0.342334596 |
| 16911 | LIM domain only 4 | 0.032416988 | -0.342185039 |
| 244672 | CWF19-like 2, cell cycle control (S. pombe) | 0.083720982 | -0.34210524 |
| 13002 | DnaJ (Hsp40) homolog, subfamily C, member 5 | 0.054644073 | -0.341152273 |
| 52055 | RAB11 family interacting protein 5 (class I) | 0.092298595 | -0.34067296 |
| 50528 | transmembrane protease, serine 2 | 0.02003218 | -0.340167183 |
| 240753 | pleckstrin homology domain containing, family A member 6 | 0.041664973 | -0.33890591 |
| 14537 | glucosaminyl (N-acetyl) transferase 1, core 2 | 0.02258937 | -0.337407599 |
| 17356 | myeloid/lymphoid or mixed-lineage leukemia (trithorax homolog, Drosophila); translocated to, 4 | 0.052656515 | -0.335209282 |
| 105239 | ring finger protein 44 | 0.051904298 | -0.333397028 |
| 12050 | BCL2-like 2 | 0.003584395 | -0.333170511 |
| 52076 | transmembrane protein 38B | 0.062442359 | -0.33277164 |
| 226747 | AT hook containing transcription factor 1 | 0.030313851 | -0.331400173 |
| 66855 | transcription factor 25 (basic helix-loop-helix) | 0.054570083 | -0.328362646 |
| 53334 | golgi SNAP receptor complex member 1 | 0.054570083 | -0.326725764 |
| 216991 | ArfGAP with dual PH domains 2 | 0.021754619 | -0.326499735 |
| 16593 | kinesin light chain 1 | 0.023675578 | -0.326157367 |
| 68525 | Ellis van Creveld syndrome 2 | 0.00051341 | -0.325138015 |
| 71704 | Rho guanine nucleotide exchange factor (GEF) 3 | 0.054644073 | -0.324984329 |
| 17434 | molybdenum cofactor synthesis 2 | 0.048635614 | -0.323103196 |
| 54357 | erythrocyte protein band 4.1-like 4b | 0.054570083 | -0.322270285 |
| 100046003 | 100046003 | 0.018196193 | -0.322225477 |
| 14009 | ets variant 1 | 0.052091205 | 0.32325376 |
| 66218 | NADH dehydrogenase (ubiquinone) 1 beta subcomplex, 9 | 0.029722703 | 0.323999288 |
| 100048508 | 100048508 | 0.036631652 | 0.324246379 |
| 73047 | calcium/calmodulin-dependent protein kinase II inhibitor 2 | 0.053130544 | 0.326341957 |
| 100044842 | 100044842 | 0.059039923 | 0.327149782 |
| 67903 | GIPC PDZ domain containing family, member 1 | 0.008901945 | 0.327191145 |
| 66616 | sorting nexin 9 | 0.036164118 | 0.327664678 |
| 59047 | polynucleotide kinase 3'- phosphatase | 0.054662046 | 0.327962243 |
| 66387 | nudix (nucleoside diphosphate linked moiety X)-type motif 8 | 0.057152277 | 0.328654258 |
| 66268 | phosphatidylinositol glycan anchor biosynthesis, class Y-like | 0.052826034 | 0.329609218 |
| 17122 | Max dimerization protein 4 | 0.091738738 | 0.329764305 |
| 234388 | coiled-coil domain containing 124 | 0.053224195 | 0.331072864 |
| 54397 | palmitoyl-protein thioesterase 2 | 0.018196193 | 0.336428874 |
| 28193 | receptor accessory protein 3 | 0.036631652 | 0.336755494 |
| 11818 | apolipoprotein H | 0.075013136 | 0.336896907 |
| 100047905 | 100047905 | 0.09607484 | 0.33896189 |
| 230696 | expressed sequence AU022252 | 0.085862447 | 0.342443193 |
| 381801 | TatD DNase domain containing 2 | 0.00995078 | 0.345877717 |
| 109305 | ORAI calcium release-activated calcium modulator 1 | 0.051933897 | 0.347656066 |
| 19341 | RAB4A, member RAS oncogene family | 0.037185116 | 0.348176908 |
| 101122 | RNA pseudouridylate synthase domain containing 3 | 0.053662272 | 0.349698539 |
| 20322 | sorbitol dehydrogenase | 0.064605437 | 0.350186913 |
| 240539 | ArfGAP with RhoGAP domain, ankyrin repeat and PH domain 2 pseudogene | 0.006148092 | 0.352926328 |
| 232337 | zinc finger protein 637 | 0.051904298 | 0.352997175 |
| 67994 | mitochondrial ribosomal protein S11 | 0.064605437 | 0.355451187 |
| 326619 | histone cluster 1, H4a | 0.035793129 | 0.355952701 |
| 18643 | profilin 1 | 0.023675578 | 0.359113495 |
| 66923 | polybromo 1 | 0.008901945 | 0.3635696 |
| 26426 | nucleotide binding protein 2 | 0.091738738 | 0.365570436 |
| 100044298 | 100044298 | 0.065126671 | 0.36724496 |
| 216169 | abhydrolase domain containing 17A | 0.09607484 | 0.373753333 |
| 104771 | JNK1/MAPK8-associated membrane protein | 0.008901945 | 0.374201488 |
| 68440 | dual specificity phosphatase 23 | 0.044549406 | 0.374998345 |
| 12162 | bone morphogenetic protein 7 | 0.016616843 | 0.376937144 |
| 20054 | ribosomal protein S15 | 0.052091205 | 0.379131775 |
| 319165 | histone cluster 1, H2ad | 0.055423347 | 0.383125509 |
| 66496 | pancreatic progenitor cell differentiation and proliferation factor homolog (zebrafish)RIKEN cDNA 2700038C09 gene | 0.053041848 | 0.384796821 |
| 22057 | transducer of ErbB-2.1 | 0.021585686 | 0.385369525 |
| 66416 | NADH dehydrogenase (ubiquinone) 1 alpha subcomplex, 7 (B14.5a) | 0.058744814 | 0.385794512 |
| 68338 | golgi transport 1 homolog A (S. cerevisiae) | 0.054662046 | 0.387877384 |
| 319168 | histone cluster 1, H2ah | 0.015860637 | 0.389408424 |
| 107686 | small nuclear ribonucleoprotein D2 | 0.091738738 | 0.390429566 |
| 621893 | histone cluster 2, H2ab | 0.009770442 | 0.391346834 |
| 19383 | hnRNP-associated with lethal yellow | 0.00075628 | 0.391474473 |
| 73737 | RIKEN cDNA 1110008P14 gene | 0.032677371 | 0.398014488 |
| 68089 | actin related protein 2/3 complex, subunit 4 | 0.02003218 | 0.399036262 |
| 319169 | histone cluster 1, H2ak | 0.013478517 | 0.401665212 |
| 28064 | Yip1 domain family, member 3 | 0.044530499 | 0.401964356 |
| 100048301 | 100048301 | 0.011854138 | 0.403715376 |
| 100046746 | 100046746 | 0.054570083 | 0.40444175 |
| 66056 | zinc finger protein 524 | 0.022364842 | 0.407015501 |
| 50799 | solute carrier family 25 (mitochondrial carrier, adenine nucleotide translocator), member 13 | 0.064605437 | 0.407090576 |
| 215210 | transmembrane protein 120A | 0.022098202 | 0.407249254 |
| 18789 | poly (A) polymerase alpha | 0.014581131 | 0.419239362 |
| 19211 | phosphatase and tensin homolog | 0.019548933 | 0.420604476 |
| 677180 | 677180 | 0.074617699 | 0.426572257 |
| 208092 | charged multivesicular body protein 6 | 0.065838242 | 0.430227264 |
| 668300 | ribosomal protein, large, P0, pseudogene 1 | 0.055196775 | 0.431237311 |
| 269336 | coiled-coil domain containing 32 | 0.023675578 | 0.43705256 |
| 66508 | late endosomal/lysosomal adaptor, MAPK and MTOR activator 1 | 0.035686973 | 0.439381441 |
| 16624 | kallikrein 1-related peptidase b8 | 0.065297573 | 0.446476173 |
| 66256 | signal sequence receptor, beta | 0.072643268 | 0.447264318 |
| 22142 | tubulin, alpha 1A | 0.091031093 | 0.448637506 |
| 68043 | N-6 adenine-specific DNA methyltransferase 2 (putative) | 0.056456445 | 0.449339915 |
| 59038 | peroxisomal membrane protein 4 | 0.02003218 | 0.459298284 |
| 16149 | CD74 antigen (invariant polypeptide of major histocompatibility complex, class II antigen-associated) | 0.029722703 | 0.459448612 |
| 13864 | nuclear receptor subfamily 2, group F, member 6 | 0.059448689 | 0.459749988 |
| 246221 | mercaptopyruvate sulfurtransferase | 0.022364842 | 0.462300554 |
| 100048733 | 100048733 | 0.046566126 | 0.473229941 |
| 77254 | Yip1 interacting factor homolog B (S. cerevisiae) | 0.033994413 | 0.485767168 |
| 414077 | WD repeat domain 83 opposite strand | 0.016313056 | 0.48674867 |
| 20657 | superoxide dismutase 3, extracellular | 0.086625488 | 0.492735345 |
| 71755 | dihydrodiol dehydrogenase (dimeric) | 0.056573036 | 0.50645056 |
| 18813 | proliferation-associated 2G4 | 0.092703823 | 0.506597333 |
| 11421 | angiotensin I converting enzyme (peptidyl-dipeptidase A) 1 | 0.036631652 | 0.514530135 |
| 66847 | histidine triad nucleotide binding protein 3 | 0.088456497 | 0.515057588 |
| 14635 | galactokinase 1 | 0.053088348 | 0.522344807 |
| 67880 | dicarbonyl L-xylulose reductase | 0.060946364 | 0.52435788 |
| 15461 | Harvey rat sarcoma virus oncogene | 0.058854151 | 0.537457064 |
| 16403 | integrin alpha 6 | 0.028677146 | 0.559391688 |
| 108159 | UBX domain protein 8 | 0.053088348 | 0.561221158 |
| 216185 | predicted gene 4799 | 0.083094346 | 0.597180123 |
| 320415 | GTP cyclohydrolase I feedback regulator | 0.040033555 | 0.598153612 |
| 109154 | malectin | 0.099195851 | 0.601375608 |
| 78928 | phosphatidylinositol glycan anchor biosynthesis, class T | 0.001244111 | 0.679984632 |
